# Can yStage Ⅰ/Ⅱ rectal cancer patients be treated in the same way as stage Ⅰ/Ⅱ patients?

**DOI:** 10.1016/j.heliyon.2024.e39530

**Published:** 2024-10-18

**Authors:** Shumpei Mukai, Naruhiko Sawada, Yusuke Takehara, Kenta Nakahara, Yuta Enami, Fumio Ishida, Shin-ei Kudo

**Affiliations:** Digestive Disease Center, Showa University, Northern Yokohama Hospital, Yokohama, Japan

**Keywords:** Local advanced rectal cancer, Neoadjuvant therapy, Adjuvant therapy, yStage Ⅱ, yStage Ⅰ, Recurrence risk factors, Long-term prognosis

## Abstract

**Background:**

Neoadjuvant therapy (NAT) before radical surgery are effective treatments for locally advanced rectal cancer. However, the treatment strategy after NAT and surgery is still unclear. It is difficult to accurately evaluate the stage before NAT, as some cases are downstaged by NAT.

**Objective:**

We investigated the treatment strategies based on the postoperative pathology of patients with yStage Ⅰ or Ⅱ rectal cancer who underwent NAT and radical resection.

**Design:**

They patients were retrospectively evaluated the long-term outcomes. They were divided into patients with yStage I/II receiving NAT and patients with stage I/II patients without NAT (non-NAT). Disease-free survival (DFS) and overall survival (OS) were examined, and the prognosis was compared. Cox proportional hazard model was used to examine the recurrence risk factors in all patients or NAT. We compared the effects of adjuvant therapy in NAT.

**Patients:**

Overall, 521 patients histologically diagnosed with yStage I/II or stage I/II who underwent surgery for rectal cancer between April 2001 and July 2019 were eligible.

**Results:**

The NAT and non-NAT groups included 80 and 441 patients, respectively. DFS was significantly lower in NAT, but there was no difference in OS between the two groups. All patients had several recurrence risk factors, but none of the NAT had such risk factors. No significant difference in DFS and OS was found between NAT with and without adjuvant chemotherapy.

**Limitation:**

This is a single-center retrospective study.

**Conclusions:**

NAT had lower DFS than non-NAT, but no difference in OS was observed. No significant recurrence risk factors were observed in NAT. Adjuvant chemotherapy for NAT may have no benefit.

## Highlights

•Postoperative treatment strategy for rectal cancer remains to be determined.
•Long-term prognosis of yStage I/II rectal cancer patients was investigated.
•Recurrence risk factors for yStage I/II or Stage I/II rectal cancer were examined.
•Effects of adjuvant therapy in yStage I/II rectal cancer patients were assessed.

## 1 Introduction

Neoadjuvant therapy (NAT), such as radiotherapy, chemotherapy, or chemoradiotherapy (CRT) before radical surgery is one of the effective treatments for locally advanced rectal cancer. NAT reduces local recurrence [[1], [2], [3], [4]]. However, there are no conclusions about the treatment strategy after NAT and radical surgery. Adjuvant chemotherapy after NAT and radical surgery has been reported to be effective [[5], [6], [7], [8], [9]], whereas others reported that it is not effective [[10], [11], [12], [13], [14]] and its usefulness is unknown. In rectal cancer, adjuvant chemotherapy is recommended for stage III or high-risk stage Ⅱ cases [15] but not for stage I cases or low-risk stage II cases. However, it is unclear whether it can be applied to patients after NAT. Moreover, no conclusion has been reached on the treatment strategy when cStage III is downstaged to yStage I or II by NAT. The effect of NAT varies among patients. It remains unclear whether the initial clinical or pathological stage should be used to determine the risk/benefit of adjuvant treatment [15]. Additionally, accurate evaluation of lymph node metastasis before NAT is difficult. Pathologic assessment of lymph node metastasis can only be diagnosed after surgery. Herein, the yStage and stage or pStage were defined as the histopathological stages with and without NAT, and cStage was defined as the stage estimated from preoperative colonoscopic and imaging findings. We investigated the treatment strategies based on the postoperative pathology of patients with yStage Ⅰ or Ⅱ rectal cancer who underwent NAT and radical resection. The purpose of this study is to evaluate whether patients with yStage I or II can be treated similarly to patients with stage I or II. They were compared in a retrospective cohort. According to European Society for Medical Oncology (ESMO)'s clinical practice guidelines, postoperative pathological staging (ypTNM) can predict a high risk of subsequent local and distant recurrences [15]. However, no reports comparing the prognosis of patients with yStage I or II with that of patients with stage I or II were found on Pubmed searches using keywords such as rectal cancer, NAT, N0, yN0, yStage I, and yStage II.

## 2 Materials and methods

This single-center retrospective study enrolled patients who underwent rectal resection or rectal amputation for Ra and/or Rb rectal cancer at our department between April 1, 2001, and July 31, 2019. Patients were histologically confirmed to have adenocarcinoma with T1–4, N0, and M0. Tumors were staged according to the TNM Classification of Malignant Tumors, 8th edition. Patients were excluded if they had previously had other cancers and ulcerative colitis. HNPCC, recurrent, or Cur B/C cases were excluded.

The patients with rectal cancer were divided into yStage I or II (ypT1–4, ypN0, yM0) patients with neoadjuvant therapy and radical surgery (NAT) and stage I or II (pT1–4, pN0, M0) patients with radical surgery but without NAT (non-NAT). The prognosis of NAT and non-NAT was compared. The recurrence risk factors in all patients or NAT were evaluated. NAT was divided into the patients with adjuvant chemotherapy (AT) and the patients without adjuvant chemotherapy (non-AT), and their prognosis was compared.

### 2.1 Neoadjuvant therapy

NAT (CRT, chemotherapy, or radiotherapy) was performed for rectal cancer patients suspected as having T3 or deeper or lymph node metastasis before treatment.

### 2.2 Surgery

Rectal resection, rectal amputation, or total pelvic exenteration with total mesorectal excision and D2 or D3 lymphadenectomy were performed.

### 2.3 Adjuvant therapy

Adjuvant therapy was performed for the patients with recurrence risk factors, such as intestinal obstruction, invasion depth T4, lymphatic invasion, venous invasion, nerve invasion, undifferentiated carcinoma (including components of mucinous carcinoma and poorly differentiated adenocarcinoma), and <12 dissected lymph nodes, high carcinoembryonic antigen (CEA) level.

### 2.4 Follow-up

Follow-up examinations included medical history taking, physical examination, serum carcinoembryonic antigen measurement, lower gastrointestinal endoscopy, and chest–abdominal–pelvic computed tomography (CT) were performed with an interval of 3–6 months.

### 2.5 Statistical analysis

Continuous data were reported as mean and standard deviation. Student's t-test was used for analyzing normal continuous data. Chi-square test was used for examining categorical data. P-value <0.05 determined by two-tailed test was considered statistically significant. All statistical analyses were performed by JMP® Pro (version 16.0.0, SAS Institute Inc., Cary, NC; 2021). Disease-free survival (DFS), local recurrence-free survival (LRFS), and overall survival (OS) were defined as the time from surgery to the first recurrence (local or distant metastasis), from surgery to the first local recurrence, and from surgery to death from any cause, respectively.

Regression analysis was performed on all patients to determine the odds ratios of recurrence for various factors, and NAT and non-NAT backgrounds were adjusted by 1:1 propensity-score matching. It was adjusted for recurrence as outcome and age, sex, surgical procedure (APR or TP vs. others), bowel obstruction, pathological T classification, lymphatic invasion, venous invasion, undifferentiated cancer, number of lymph nodes dissected, CEA, and adjuvant therapy as covariates. DFS, LRFS, and OS were evaluated in NAT and non-NAT groups. The DFS, LRFS, and OS survival curves were estimated using the Kaplan–Meier method and compared with log-rank tests. We also examined the 5-year DFS, LRFS, and OS rates. The recurrence risk factors were evaluated in all patients or NAT. Hazard ratios were calculated using the Cox proportional hazard ratio model in the multivariate analysis. Age, sex, surgical procedure, bowel obstruction, T4 invasion depth, lymphatic invasion, venous invasion, undifferentiated cancer, <12 dissected lymph nodes, high CEA, NAT, and adjuvant therapy were selected as factors for the Cox proportional hazards model. NAT was further divided into AT and non-AT subgroups. Their DFS, LRFS, and OS survival curves were estimated using the Kaplan–Meier method and compared with log-rank tests.

The present study was approved by the Ethics Committee of Showa University, and patient informed consent was obtained through the opt-out method. This study followed STROBE guidelines.

## 3 Results

**Fig. 1:**
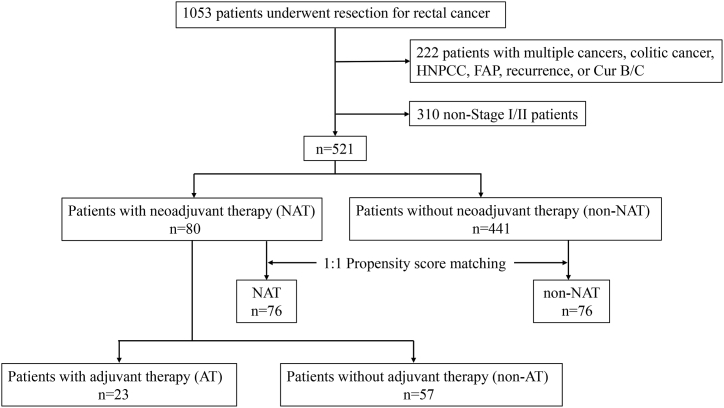
Flow diagram describing patient selection and exclusion.

In total, 1053 patients underwent resection for rectal cancer. Moreover, 97 patients with multiple cancers, 8 with colitic cancer, 11 with HNPCC, 1 with FAP, 15 with recurrence, and 90 with Cur B/C were excluded; 310 patients with nonStage I/II were also excluded. Ultimately, 521 patients (331 men and 190 women) were examined (Fig. 1). Patients’ mean and median ages were 63.9 (SD 11.6) and 65 years, respectively. The mean and median follow-up times were 68.6 (SD 33.4), and 64.0 months, respectively. The NAT and non-NAT included 80 and 441 cases, respectively.

### 3.1 Matching data sources and propensity scores

Patients’ background characteristics are shown in Table 1. Differences were noted in the rates of surgical procedure (p < 0.001), intestinal obstruction (p = 0.009), lymphatic invasion (p < 0.001), venous invasion (p = 0.002), <12 lymph nodes dissected (p = 0.045), and CEA (p = 0.014) between the NAT (n = 80) and non-NAT (n = 441) groups. Regression analysis was performed for all patients. The odds ratio for recurrence was increased for surgical procedure (p = 0.003), lymphatic invasion (p = 0.034), venous invasion (p = 0.008), and lymph node dissection <12 (p = 0.029) (Table 2). Therefore, a propensity-score model was run to achieve a balanced distribution of these baseline covariates (ratio 1:1). After propensity-score matching, no significant differences were observed in the results of pairwise comparisons of all covariates between the NAT (n = 76) and non-NAT (n = 76) (Table 1).

**Table 1:** Patient characteristics before and after propensity-score matching for patients with and without neoadjuvant therapy.

	NAT	non-NAT		NAT	non-NAT	
	(n = 80)	(n = 441)	P-value	(n = 76)	(n = 76)	P-value
Age (years), mean (SD)	62.3 (10.5)	64.2 (11.8)	0.186	62.6 (10.6)	61.0 (12.3)	0.41
Sex ratio (M:F)	57:23:00	274:167	0.114	53:23:00	57:19:00	0.468
APR, TP (Operation)						
Yes	23 (28.8)	49 (11.1)	<0.001	20 (26.3)	19 (25.0)	0.853
No	57 (71.3)	392 (88.9)		56 (73.7)	57 (75.0)	
Obstruction						
Yes	8 (10.1)	13 (3.0)	0.009	6 (7.9)	3 (4.0)	0.298
No	71 (89.9)	428 (97.1)		70 (92.1)	73 (96.1)	
Pathological T classification						
T4	1 (1.3)	8 (1.8)	0.71	1 (1.3)	0 (0.0)	0.238
≤3	79 (98.8)	433 (98.2)		75 (98.7)	76 (100.0)	
Lymphatic invasion						
Yes	8 (10.0)	148 (33.6)	<0.001	8 (10.5)	3 (4.0)	0.111
No	72 (90.0)	293 (66.4)		68 (89.5)	73 (96.1)	
Vascular invasion						
Yes	35 (43.8)	277 (62.8)	0.002	34 (44.7)	32 (42.1)	0.743
No	47 (56.3)	164 (37.2)		42 (55.3)	44 (57.9)	
Tumor differentiation						
Poor	13 (16.3)	60 (13.6)	0.538	12 (15.8)	10 (13.2)	0.645
Well to moderate	67 (83.8)	381 (86.4)		64 (84.2)	66 (86.8)	
Number of lymph node resection						
<12	27 (33.8)	108 (24.5)	0.089	26 (34.2)	22 (29.0)	0.485
≥12	53(66.3)	333 (75.5)		50 (65.8)	54 (71.1)	
CEA (ng/mL)						
>5 ng/mL	17 (21.3)	46 (10.4)	0.011	15 (19.7)	12 (15.8)	0.524
≤5 ng/mL	63 (78.8)	395(89.6)		61 (80.3)	64 (84.2)	
Adjuvant therapy						
Yes	23 (28.8)	89 (20.2)	0.095	20 (26.3)	15 (79.7)	0.335
No	57 (71.3)	352 (79.8)		56 (73.7)	61 (80.3)	
NAT, yStage I or II patients with neoadjuvant therapy; non-NAT, stage I or II patients without neoadjuvant therapy; APR, abdominoperineal resection; TP, total pelvic exenteration; CEA, carcinoembryonic antigen. The analysis was adjusted for recurrence as outcome and age, sex, operation, bowel obstruction, pathological T classification, lymphatic invasion, venous invasion, tumor differentiation, number of lymph node resection, CEA, and adjuvant therapy as covariates. Values are n (%) unless otherwise indicated.

**Table 2:** Risk factors for recurrence in all patients.

	Odds ratios	95%CI	p-value
Age (≥65 years)	1.322	0.714–2.446	0.374
Male	1.378	0.717–2.649	0.336
APR, TP	3.073	1.479–6.384	0.003
LAP	0.560	0.280–1.120	0.101
Obstraction	3.078	0.932–10.165	0.065
T4	4.859	0.902–26.174	0.066
lymphatic invasion	2.080	1.055–4.101	0.034
vascular invasion	2.702	1.300–5.615	0.008
Poorly tumor differentiation	1.212	0.521–2.821	0.656
number of lymph node resection (<12)	2.081	1.076–4.025	0.029
CEA (>5 ng/mL)	0.991	0.416–2.357	0.983
Neoadjuvant therapy			
CRT/none	2.168	0.911–5.160	0.080
CRT/CT	0.451	0.049–4.108	0.480
CRT/RT	1.248	0.098–15.867	0.864
CT/none	4.811	0.536–43.219	0.161
CT/RT	2.770	0.104–73.469	0.542
RT/none	1.737	0.148–20.338	0.660
Adjuvant therapy			
Ox/Non-Ox	1.932	0.318–11.726	0.474
Ox/nonCT	2.011	0.366–11.040	0.421
Non-Ox/nonCT	1.041	0.495–2.190	0.916
Odds ratios for recurrence were calculated using regression analysis. Values in parentheses are 95 % confidence intervals. APR, abdominoperineal resection; TP, total pelvic exenteration; LAP, laparoscopic surgery; CEA, carcinoembryonic antigen; CRT, chemoradiotyherapy; CT, chemotherapy; RT, radiotherapy; none, no neoadjuvant therapy; Ox, oxaliplatin-based chemothetapy; Non-Ox, nonoxaliplatin-based chemotherapy; nonCT, no chemotherapy.			

### 3.2 Therapy

Table 3 shows the details of NAT, operation, and adjuvant therapy. In the matched patients, the NATs included CRT in 64 cases, chemotherapy in 7, and radiation therapy (RT) in 5. In the CRT or RT group, 45.6–50.8 Gy irradiation was administered. One patient each discontinued S-1 on CRT and CRT. Altogether, 437 LAR, 7 HAR, 70 APR, 5 Hartmann, and two total pelvic exenterations were performed (Table 3). AT was administered to 23 patients (Table 3). AT was continued for ≥3 months in 18 (78.3 %) patients.

**Table 3:** Treatment for neoadjuvant therapy, surgery, and adjuvant therapy.

	Before matching		After matching	
	NAT	non-NAT	NAT	non-NAT
	(n = 82)	(n = 441)	(n = 76)	(n = 76)
Neoadjuvant therapy				
CRT	68 (85.0)		64 (84.2)	
RT + S-1	47 (58.8)		43 (56.6)	
RT + Doxifluridine	11 (13.8)		11 (14.5)	
RT + CaoeOX	3 (3.8)		3 (4.0)	
RT + UFT	3 (3.8)		3 (4.0)	
RT + CapeOX + Bev	1 (1.3)		1 (1.3)	
RT + FOLFOX + Cmab	1 (1.3)		1 (1.3)	
RT + UFT/LV	1 (1.3)		1 (1.3)	
RT + 5FU	1 (1.3)		1 (1.3)	
CT	7 (8.8)		7 (9.2)	
CapeOX + Bv	4 (5.0)		4 (5.3)	
CapeOX	1 (1.3)		1 (1.3)	
FOLFOXILI	1 (1.3)		1 (1.3)	
S-1	1 (1.3)		1 (1.3)	
RT	5 (6.3)		5 (6.6)	
Operation				
LLAR	37 (46.3)	332 (75.3)	36 (47.4)	51 (67.1)
OLAR	19 (23.8)	49 (11.1)	19(250	3 (4.0)
LHAR	0 (0.0)	7 (1.6)	0 (0.0)	1 (1.3)
LAPR	17 (21.3)	29 (6.6)	14 (18.4)	10 (13.2)
OAPR	5 (6.3)	19 (4.3)	5 (6.6)	9 (11.8)
LHRT	1 (1.3)	2 (0.5)	1 (1.3)	1 (1.3)
OHRT	0 (0.0)	2 (0.5)	0 (0.0)	1 (1.3)
TP	1 (1.3)	1 (0.2)	1 (1.3)	0 (0.0)
Adjuvant therapy				
CAPOX	8 (10.0)	0 (0.0)	7 (9.2)	0 (0.0)
Cape	3 (3.8)	2 (0.5)	3 (4.0)	0 (0.0)
UFT/LV	7 (8.8)	27 (6.1)	5 (6.6)	2 (2.6)
UFT	2 (2.5)	36 (8.2)	2 (2.6)	9 (11.8)
S-1	1 (1.3)	10 (2.3)	1 (1.3)	2 (2.6)
Doxifluridine	0 (0.0)	7 (1.6)	0 (0.0)	1 (1.3)
FOLFOX	2 (2.5)	1 (0.2)	2 (2.6)	1 (1.3)
5-FU/LV	0 (0.0)	3 (0.7)	0 (0.0)	0 (0.0)
5-FU	0 (0.0)	3 (0.7)	0 (0.0)	0 (0.0)
unknown	0 (0.0)	3 (0.7)	0 (0.0)	1 (1.3)
No	57 (71.3)	349 (79.1)	56 (73.7)	60 (79.0)
Values are n (%) unless otherwise indicated. NAT, yStage I or II patients with neoadjuvant therapy; non-NAT, stage I or II patients without neoadjuvant therapy; CRT, chemoradiation therapy; CT, chemotherapy; RT, radiation therapy; LLAR, laparoscopic low anterior resection; OLAR, open low anterior resection; LHAR, laparoscopic high anterior resection; LAPR, laparoscopic abdominoperineal resection; OAPR, open abdominoperineal resection; LHRT, laparoscopic Hartmann's operation; OHRT, open Hartmann's operation; TP, total pelvic exenteration				

### 3.3 Comparison of DFS and OS between the groups with and without preoperative treatment

**Fig. 2:**
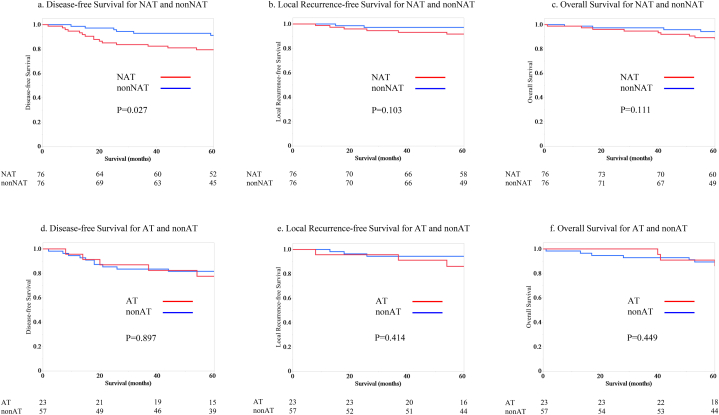
Kaplan–Meier curves for disease-free and overall survival a, Disease-free survival for yStage I or II patients with neoadjuvant therapy (NAT) and stage I or II patients without neoadjuvant therapy (non-NAT). b, Local Recurrence-free Survival for NAT and non-NAT. c, Overall survival for NAT and non-NAT. d, Disease-free survival for yStage I or II patients with (AT) and without adjuvant chemotherapy (non-AT). e, Local Recurrence-free Survival for AT and non-AT. f, Overall survival for AT and non-AT.

NAT had significantly lower DFS than non-NAT (log-rank test p = 0.027) (Fig. 2a). The 5-year DFS rates were 79.4 % for NAT and 91.1 % for non-NAT. No significant difference in LRFS was found between the NAT and non-NAT groups (p = 0.103) (Fig. 2b). The 5-year LRFS rates were 91.7 % for NAT and 97.1 % for non-NAT. No significant difference in OS was noted between the NAT and non-NAT groups (p = 0.111) (Fig. 2c). The 5-year OS rates were 87.8 % for NAT and 94.2 % for non-NAT.

### 3.4 Comparison of recurrence risk factors

Cox proportional hazards analysis revealed that APR or TP (p = 0.001), bowel obstruction (p = 0.007), and vascular invasion (p = 0.004) were risk factors for recurrence in all patients (Table 4). No significant recurrence risk factors were found in NAT (Table 4).

**Table 4:** Risk factors for recurrence in all patients and yStage I or II patients with neoadjuvant therapy.

	All patients (n = 521)		NAT (n = 80)	
	Hazard ratio	P-value	Hazard ratio	P-value
Age (≥65 years)	1.282 (0.747, 2.199)	0.368	1.341 (0.452, 3.981)	0.597
Male	1.390 (0.770, 2.511)	0.275	3.363 (0.727, 15.549)	0.121
APR, TP	2.951 (1.579, 5.516)	0.001	0.535 (0.121, 2.367)	0.409
Obstruction	3.759 (1.443, 9.788)	0.007	2.099 (0.414, 10.633)	0.37
T4	3.284 (0.832, 12.949)	0.09	6.757e-9 (0.000, -)	1
lymphatic invasion	1.773 (0.980, 3.210)	0.058	0.509 (0.053, 4.906)	0.559
vascular invasion	2.666 (1.369, 5.190)	0.004	2.832 (0.884, 9.070)	0.08
Poorly tumor differentiation	1.388 (0.653, 2.951)	0.394	0.419 (0.051, 3.411)	0.416
number of lymph node resection (<12)	1.724 (0.965, 3.080)	0.066	1.804 (0.504, 6.455)	0.365
CEA (>5 ng/mL)	1.169 (0.543, 2.514)	0.69	0.501 (0.103, 2.426)	0.39
Neoadjuvant therapy	1.959 (0.979, 3.917)	0.057		
Adjuvant therapy	1.099 (0.606, 1.994)	0.757	1.580 (0.491, 5.092)	0.443
The Cox proportional hazards model was used to evaluate the recurrence risk factors. Values in parentheses are 95 % confidence intervals. NAT, yStage I/II patients with neoadjuvant therapy; APR, abdominoperineal resection; TP, total pelvic exenteration; CEA, carcinoembryonic antigen.				

### 3.5 Comparison of DFS and OS between patients with and without postoperative adjuvant chemotherapy in NAT

The characteristics of AT (n = 23) and non-AT (n = 57) showed no significant difference except for age (Table 5). Moreover, no difference was observed in the DFS (log-rank test p = 0.897) (Fig. 2d), LRFS (p = 414) (Fig. 2e), and OS (p = 0.449) (Fig. 2f) between the AT and non-AT groups. The 5-year DFS rates were 77.5 % for AT and 81.6 % for non-AT. The 5-year LRFS rates were 86.0 % for AT and 94.4 % for non-AT. The 5-year OS rates were 85.9 % for AT and 89.3 % for non-AT.

**Table 5:** Characteristics of yStage I or II patients with and without adjuvant chemotherapy.

	AT	non-AT	P-value
	(n = 23)	(n = 57)	
Age (years), mean (SD)	59.0 (10.3)	63.7 (10.3)	0.066
Sex ratio (M:F)	15:08	42:15:00	0.454
APR, TP (Operation)			
Yes	8 (34.8)	15 (26.3)	0.454
No	15 (65.2)	42 (73.7)	
Obstruction			
Yes	3 (13.6)	5 (8.8)	0.532
No	19 (86.4)	52 (91.2)	
Pathological T classification			
T4	0 (0.0)	1 (1.8)	0.409
≤3	23 (100.0)	56 (98.3)	
Lymphatic invasion			
Yes	1 (4.4)	7 (12.3)	0.25
No	22 (95.7)	50 (87.7)	
Vascular invasion			
Yes	8 (34.8)	27 (47.4)	0.301
No	15 (65.2)	30 (52.6)	
Tumor differentiation			
Poor	6 (26.1)	7 (12.3)	0.143
Well to moderate	17 (73.9)	50 (87.7)	
Number of lymph node resection			
<12	7 (30.4)	20 (35.1)	0.689
≥12	16 (69.6)	37 (64.9)	
CEA (ng/mL)			
>5 ng/mL	4 (17.4)	13 (22.8)	0.587
≤5 ng/mL	19 (82.6)	44 (77.2)	
Values are n (%) unless otherwise indicated. AT, yStage I or II patients with adjuvant therapy; non-AT, yStage I or II patients without adjuvant therapy; APR, abdominoperineal resection; TP, total pelvic exenteration; CEA, carcinoembryonic antigen.			

## 4 Discussion

Radical surgery after preoperative treatments, such as radiochemotherapy, radiotherapy, and chemotherapy, is one of the effective treatment methods for locally advanced rectal cancer. However, no conclusions have been reached regarding treatment strategies after radical surgery. The ESMO guidelines state that considering adjuvant chemotherapy for patients with “high-risk” yp stage II is reasonable, and the level of scientific evidence for sufficient benefit is much lower than for colon cancer [15]. The ASCO guidelines do not address the management of patients with yStage I/II rectal cancer [16]. This study aimed to assess whether patients with yStage I or II can be observed like those in patients with stage I or II or whether they should be treated with adjuvant chemotherapy as patients with stage III. Therefore, this study compared the long-term prognosis of patients with yStage I or II with that of patients with stage I or II. Differences in patient characteristics and factors that may affect recurrence risk were noted (Table 2); however, these were balanced by propensity-score matching (Table 1). NAT had lower DFS than non-NAT, with no significant difference in OS. Of 76 patients with NAT, 16 experienced recurrence. Their mean and median survival times were 73.4 (8.8) and 72.0 months, respectively. In other words, patients could survive for a long time even if they relapsed; thus, no significant difference was noted in OS. Although a significant difference was observed in DFS, none was observed in LRFS, potentially because NAT suppressed local recurrence [[1], [2], [3], [4]].

An accurate evaluation of the degree of progression before preoperative treatment is difficult. The ESMO guidelines recommend that preoperative diagnosis of the depth of rectal cancer should be made by endoscopic ultrasound (EUS) or magnetic resonance imaging (MRI). The combined use of CT and MRI indicates that clinical nodal staging is unreliable [15]. According to a meta-analysis by Marone et al., EUS demonstrated an accuracy rate of 63%–96 % for the depth of invasion [17]. In the preoperative diagnosis of lymph nodes by high-resolution MRI, accuracy rates of 74 % and 85 % were reported by Akasu et al. [18] and Brown et al. [19], respectively. There were 2 cStage IV cases, 52 cStage III cases, and 26 cStage II cases before preoperative therapy in NAT. There were 33 cStage III cases, 38 stage II cases, and 9 stage I cases after preoperative therapy before operation in NAT. There were 45 pStage II cases, 35 pStage I cases after operation in NAT. There were 1 cStage IV case, 89 cStage III cases, 103 stage II cases, and 248 stage I cases before operation in non-NAT. There were 145 pStage II cases and 296 pStage I cases after operation in non-NAT. The concordance rate between preoperative cStage and postoperative pStage was 32.5 % for NAT and 67.6 % for non-NAT and was significantly lower for NAT (p < 0.0001). This is assumed to be because lymph nodes that are significantly swollen before preoperative therapy are deemed positive for lymph node metastasis even if they shrink unless they disappear. cStage downstaged 37.5 % of patients, while pStage downstaged of 83.8 % patients. Thus, the difference between the preoperative cStage and the postoperative pStage is substantial. If the postoperative pathological diagnosis is yStage I or II, it is difficult to determine whether the stage was the same before preoperative treatment or the result of downstaging of stage III. Since the recurrence rate was higher with NAT than with non-NAT, it is speculated that some cases were downstaged due to preoperative treatment, but there is no way to identify them. If the true progression before preoperative treatment was stage I or low-risk stage II, even if preoperative treatment is unavoidable due to the difficulty of diagnosis, postoperative adjuvant chemotherapy may result in overtreatment. Therefore, we considered that it is necessary to consider the treatment policy when yStage I or II is diagnosed by pathological examination.

In the present study, there was no difference in OS between NAT and non-NAT, but NAT had a higher recurrence rate than non-NAT. Additionally, preoperative treatment was found as a recurrence risk factor in all patients. However, no significant recurrence risk factor was observed in NAT, indicating that careful surveillance was required for all NAT cases. The Clinical Practice Guidelines Committee of the American Society of Colon and Rectal Surgeons recommends using clinical stage to determine the surveillance regimen for locally advanced rectal cancer treated by NAT and surgery [20]. It is considered reasonable.

On the contrary, it is necessary to evaluate whether conventional postoperative AT is effective for yStage I or II cases that had undergone radical resection after NAT. In this study, there was no prognostic difference between the presence or absence of adjuvant therapy in NAT. However, it is possible that there was no significant difference due to the small number of cases analyzed, and it is necessary to estimate this. At present, treatment is based on the clinical stage; however, in the future, if it becomes possible to modify treatment plans midway based on resection specimens and other results, avoiding overtreatment and reducing medical costs may be possible.

The use of adjuvant chemotherapy after NAT and surgery for rectal cancer is controversial. Fausto et al. reported that adjuvant chemotherapy improves 5-year OS and DFS rates for patients with rectal cancer treated with surgery and neoadjuvant chemoradiotherapy [21]. Hong et al. and Song et al. reported that oxaliplatin-based adjuvant chemotherapy improved the DFS in patients with ypStages II and III [8,9]. Chen et al. and Li et al. reported that the postoperative adjuvant chemotherapy group had more improvement in OS than the observation group [5,6]. Collette et al. suggested that only patients with favorable prognosis (ypT0–2) benefit from adjuvant chemotherapy [7]. On the contrary, Bosset et al. and Chung et al. reported that adjuvant fluorouracil-based chemotherapy after preoperative radiotherapy had no effect on DFS or OS [10,14]. Lu et al. and Zhang et al. showed that patients with ypT0–2N0 rectal cancer may not benefit from adjuvant chemotherapy [11,13]. Moreover, Hu et al. demonstrated that patients with ypTis-2N0 rectal cancer may not benefit from adjuvant chemotherapy [12]. Based on these results, it is possible that chemotherapy with oxaliplatin among patients with more than a certain degree of progression is effective. Recently, studies have introduced total NAT (TNT) that involves administering radiation and systemic therapy before radical surgery for locally advanced rectal cancer [[22], [23], [24]]. However, most reports have focused on its effectiveness and adverse events. Despite reports of adjuvant chemotherapy after TNT and radical surgery [[25], [26], [27]], no relevant data demonstrate its effect after neoadjuvant chemotherapy [28]. Thus, whether adjuvant chemotherapy is effective for patients with yStage Ⅰ/Ⅱ after TNT and surgery is unclear.

In patients with recurrent colorectal cancer, resection of recurrent lesions can be expected to improve their prognosis [[29], [30], [31], [32]]. We found that yStage I or II patients have worse DFS than patients with stage I or II, but adjuvant chemotherapy may not be effective. Therefore, strict surveillance is necessary for the early detection of postoperative recurrence.

This research has several limitations. This is a single-center retrospective study. The number of cases is also small. Preoperative and postoperative treatment methods are not standardized. Moreover, we were unable to identify any recurrence risk factors for yStage I/II. The required sample size was calculated using Easy R (EZR) version 1.68 (Saitama Medical Center, Jichi Medical University, Japan) [33]; The calculation was based on a registration period of 19 years, trial period of 24 years, 5-year survival rate, non-NAT survival rate of 90 %, NAT survival rate of 80 %, alpha error of 0.05, statistical power of 0.80, and sample number ratio of 1, resulting in 84 cases of NAT and non-NAT. However, this study only compared 76 cases. If the number of cases is increased, we may be able to identify the recurrence risk factors. Once the risk of recurrence is known, it becomes clear which patients should be prioritized for surveillance. In addition, although the efficacy of adjuvant chemotherapy for yStage I/II was not proven in this study, it may be effective if the number of cases is increased. This study should be expanded to include data from multiple centers to increase the sample size and improve the generalizability of the findings. Verifying whether adjuvant chemotherapy should be performed with a regimen that conforms to stage III or with a different regimen (e.g., molecular-targeted drugs) was also necessary. A multicenter prospective comparative study with a unified regimen is desirable for these verifications. In addition, although propensity-score matching was performed to standardize patient backgrounds, the possibility of bias cannot be denied. A prospective randomized study should be conducted.

Patients with yStage Ⅰ or Ⅱ rectal cancer after NAT and radical surgery have higher recurrence rates than those not receiving such therapies, but no difference in OS was observed between them. There were no significant recurrence risk factors found in yStage Ⅰ or Ⅱ patients. Finally, yStage Ⅰ or Ⅱ patients may not benefit from AT.

## CRediT authorship contribution statement

**Shumpei Mukai:** Writing – original draft, Visualization, Software, Resources, Methodology, Investigation, Funding acquisition, Formal analysis, Data curation, Conceptualization. **Naruhiko Sawada:** Writing – review & editing, Validation, Data curation, Conceptualization. **Yusuke Takehara:** Writing – review & editing, Data curation. **Kenta Nakahara:** Writing – review & editing, Data curation. **Yuta Enami:** Writing – review & editing, Data curation. **Fumio Ishida:** Writing – review & editing, Supervision, Conceptualization. **Shin-ei Kudo:** Writing – review & editing, Supervision, Project administration, Formal analysis.

## Ethical statement

The present study was approved by the Ethics Committee of Showa University (Approval Number: 22-193-B, date of approval: November 29, 2022), and patient informed consent was obtained through the opt-out method.

## Data availability statement

The datasets generated and analyzed during the current study are available from the corresponding author on reasonable request.

## Declaration of generative AI in scientific writing

AI was not used in this study.

## Funding

This research did not receive any specific grant from funding agencies in the public, commercial, or not-for-profit sectors.

## Declaration of competing interest

The authors declare that they have no known competing financial interests or personal relationships that could have appeared to influence the work reported in this paper.
